# Scopoletin ameliorates anxiety-like behaviors in complete Freund’s adjuvant-induced mouse model

**DOI:** 10.1186/s13041-020-0560-2

**Published:** 2020-02-04

**Authors:** Li Luo, Ting Sun, Le Yang, An Liu, Qing-qing Liu, Qin-qin Tian, Yan Wang, Ming-gao Zhao, Qi Yang

**Affiliations:** 1 Precision Pharmacy & Drug Development Center, Department of Pharmacy, Tangdu Hospital, Fourth Military Medical University, Xi’an, 710038 China; 2 Department of Chemistry, School of Pharmacy, Fourth Military Medical University, Xi’an, 710032 China; 3 Department of Gastroenterology and Endoscopy Center, No. 986 Hospital, Fourth Military Medical University, Xi’an, 710054 China

**Keywords:** Anxiety, Inflammation, Glutamate, GABA, Scopoletin, Amygdala

## Abstract

Anxiety disorder is highly prevalent worldwide and represents a chronic and functionally disabling condition, with high levels of psychological stress characterized by cognitive and physiological symptoms. Scopoletin (SP), a main active compound in *Angelica dahurica*, is traditionally used for the treatment of headache, rhinitis, pain, and other conditions. Here, we evaluated the effects of SP in a mouse model of complete Freund’s adjuvant (CFA)-induced chronic inflammation anxiety. SP (2.0, 10.0, 50.0 mg/kg) administration for 2 weeks dose-dependently ameliorated CFA-induced anxiety-like behaviors in the open field test and elevated plus maze test. Moreover, we found that SP treatment inhibited microglia activation and decreased both peripheral and central IL-1β, IL-6, and TNF-α levels in a dose-dependent manner. Additionally, the imbalance in excitatory/inhibitory receptors and neurotransmitters in the basolateral nucleus after CFA injection was also modulated by SP administration. Our findings indicate that the inhibition of the nuclear factor-kappa B and mitogen-activated protein kinase signaling pathways involving anti-inflammatory activities and regulation of the excitatory/inhibitory balance can be attributed to the anxiolytic effects of SP. Moreover, our molecular docking analyses show that SP also has good affinity for gamma-aminobutyric acid (GABA) transaminase and GABA_A_ receptors. Therefore, these results suggest that SP could be a candidate compound for anxiolytic therapy and for use as a structural base for developing new drugs.

## Introduction

Anxiety disorder is highly common worldwide, with an estimated prevalence of 15% in developed countries, and seriously affects people’s life and work [1]. It is a chronic and functionally disabling condition that induces high levels of psychological stress and is characterized by cognitive symptoms, such as excessive worry and focus difficulties, as well as physiological symptoms, such as muscle tension and insomnia [1, 2]. While antidepressants and benzodiazepines are clinically useful for treating anxiety, considerable side effects, such as the risk of physical dependence, addiction, excessive sedation, and abuse, are observed in clinical practice [3, 4]. It is therefore of great significance to search for better anxiolytic drugs with fewer side effects.

Many factors, for example, danger, stress, and physical illness, can cause anxiety, but the specific pathogenesis has not been fully elucidated. Both rodent and human studies have demonstrated that inflammation plays a key role in the development of anxiety [5, 6]. In addition, increased inflammation is associated with enhanced activation of the threat- and anxiety-related brain circuitry and specifically the amygdala, which is a critical region associated with emotion and motivation in the central nervous system (CNS) [7, 8]. Among the subnuclei of the amygdala, the basolateral (BLA) nucleus regulates anxiety and related negative effects and is the primary region of information processing for cortical and thalamic afferents [9]. Moreover, the brain comprises an excitatory/inhibitory neuronal network that maintains a finely tuned balance of activity that is critical for normal function [10]. Low central gamma-aminobutyric acid (GABA) and high glutamate levels are known to result in hyperexcitation and are linked to disorders including anxiety, depression, and epilepsy [11]. However, the relationship between the excitatory/inhibitory transmission imbalance and inflammation in anxiety has not been conclusively determined.

Scopoletin (SP) is a main active compound in *Angelica dahurica*, which is a traditional Chinese herb that has been used for the treatment of headache, rhinitis, pain, and other conditions [12]. Previous studies have found that SP exhibits superoxide anion scavenging activity in the xanthine/xanthine oxidase reaction system and suppresses the differentiation of osteoclastic macrophage RAW 264.7 cells by scavenging reactive oxygen species [13, 14]. In addition, analgesic effects of SP on nociception induced by acetic acid and formalin have been reported [15, 16]. Importantly, SP can inhibit the production of pro-inflammatory cytokines such as TNF-α, IL-1β, and IL-6 in RAW 264.7 macrophages stimulated with lipopolysaccharides (LPS) [17]. Moreover, a recent study showed that SP can regulate the transcriptional level of pro-inflammatory mediators via the suppression of nuclear factor-kappa B (NF-κB) activation and the blockade of the mitogen-activated protein kinase (MAPK) signal pathway in an acute gout model [18]. SP has also shown anticonvulsant effects, both in vitro and in vivo, in a study that investigated the suppression of GABA transaminase (GABA-T) [19]. However, it is not clear whether SP can alleviate anxiety symptoms.

In this study, we therefore evaluated the effects of SP in a mouse model of chronic inflammation anxiety induced by complete Freund’s adjuvant (CFA), and investigated inflammatory responses and excitatory/inhibitory receptor and neurotransmitters levels after SP treatment to determine whether SP may be a candidate compound for the treatment of anxiety.

## Materials and methods

### Animals and treatment

Male C57BL/6 mice aged 6–8 weeks were used in this study. The animals were housed in random groups of six per cage, with food and water available ad libitum. The animals were maintained at a temperature of 24 ± 2 °C, a relative humidity of 50–60%, and under a 12-h light-dark cycle. All mice were given a commercial chow diet and allowed to adapt to the laboratory environment for at least 1 week before the experiments.

Anxiety-like behaviors were induced by an intraplantar injection of CFA (10 μL, 50% in saline, Sigma, St. Louis, MO, USA) into the plantar surface of the left hind paw of the mice. The same volume of saline (0.9%) was injected into the plantar surface of the left hind paw of control mice. SP and flumazenil (purity > 98%, Shanghai Pure One Biotechnology, China) were dissolved in 0.9% saline containing 1% dimethyl sulfoxide (DMSO). The doses of SP and flumazenil used in this study were based on previous studies [20, 21] and our preliminary tests. Animals were randomly distributed into five groups: a control group, a vehicle group, and three groups that received different doses of SP (2.0, 10.0, 50.0 mg/kg). Each group contained 12 mice. The SP groups were given intraperitoneal injections of SP after the CFA injection once a day for 14 consecutive days. The vehicle group was injected with an equal volume of 0.9% saline containing 1% DMSO at the same time. We also divided an additional cohort of mice into the following groups: control, vehicle, SP (50.0 mg/kg), and SP + flumazenil (10.0 mg/kg). Each group contained 12 mice, and the administration method was identical to the procedure described above.

### Behavioral tests

All mice were subjected to the open field test (OFT) and the elevated plus maze (EPM) test, which were conducted as described in previous reports [22]. Each mouse was brought into the testing room 2 h before the tests. Mice were administered once 30 min before the behavioral test. The OFT was always performed before the EPM, but both tests were conducted on the same day.

#### OFT

The open field (JLBehv-LAM-4, Shanghai Jiliang Software, China) was a square arena (30 × 30 × 30 cm^3^) with clear Plexiglas walls and floor, and was placed inside an isolation chamber with dim illumination and a fan. For the testing, each mouse was placed in the center of the box and allowed to freely explore for 15 min. The exploratory behaviors of the mice were recorded using a camera fixed above the chamber. The total distance traveled and time spent in the central area were analyzed using a video-tracking system (MedAssociates, St. Albans, VT, USA).

#### EPM

The apparatus (DigBehv-EPMG, Shanghai Jiliang Software) consisted of two open arms (25 × 8 × 0.5 cm^3^) and two closed arms (25 × 8 × 12 cm^3^) that extended from a common central zone (8 × 8 cm^2^). The mice were exposed to gentle handling two times to eliminate nervousness. For each test, the individual mouse was placed in the central zone facing an open arm, and allowed to explore freely for 5 min while being videotaped using a camera fixed above the maze. The time spent in and the number of entries into the open and closed arms were analyzed with a video-tracking system (MedAssociates).

### Enzyme-linked immunosorbent assay (ELISA)

Blood samples were obtained by eyeball extraction after the behavioral tests. Levels of the inflammatory cytokines IL-1β, IL-6, and TNF-α in the plasma were evaluated using ELISA kits (R&D Systems Inc., Minneapolis, MN, USA) following the manufacturer’s instructions.

### Western blot analysis

The animals were sacrificed, and the tissue samples from the bilateral BLA amygdala were dissected from brain slices under an anatomical microscope immediately after the behavioral tests. Western blot analysis was performed as previously described [23]. BLA samples were homogenized in ice-cold RIPA lysis buffer containing phosphatase and protease inhibitors. The protein content of the collected samples was quantified using the BCA protein assay. Equal amounts of protein (30 μg) were analyzed using an SDS-PAGE gel and then electro-transferred onto PVDF membranes (Invitrogen, Carlsbad, CA, USA). The following primary antibodies were used: β-actin (1:10000, Sigma), TNF-α (1:500, Abcam, Cambridge, UK), IL-6 (1:500, Abcam), IL-1β (1:500, Abcam), GluA1 (1:1000, Abcam), GluN2A (1:1000, Abcam), GluN2B (1:1000, Abcam), PSD95 (1:1000, Abcam), GABA-T (1:1000, Abcam), GABA_A_ α2 (1:1000, Abcam), GABA_A_ γ2 (1:1000, Abcam), p-p38 (1:1000, Cell Signaling Technology, Danvers, MA, USA), p38 (1:1000, Cell Signaling Technology), p-JNK (1:1000, Cell Signaling Technology), JNK (1:1000, Cell Signaling Technology), NF-κB p65 (1:1000, Cell Signaling Technology). The membranes were incubated with horseradish peroxidase-conjugated secondary antibodies (anti-rabbit/anti-mouse IgG); densitometric western blot analysis was conducted using a ChemiDoc XRS (Bio-Rad, Hercules, CA, USA) and quantified using ImageJ software (NIH, Bethesda, MD, USA). For data analysis, the band intensity of each blot was calculated as a ratio relative to that of β-actin. The intensity ratio of the control group was set at 100%, and the intensity for other treatment groups was expressed as percentages relative to the control group.

### Immunofluorescence staining

After the behavioral studies, the mice were anesthetized using pentobarbital sodium and perfused with sterile saline, followed by 4% polyformaldehyde. The separated brains were dehydrated with a sucrose gradient, 20 and 30% (w/v) sucrose in 0.1 M phosphate buffered saline (PBS) at 4 °C overnight, respectively. 20 μm-thick BLA sections were cut on a cryostat (Leica Microsystems). All sections were washed with 0.3% Triton X-100 PBS and blocked (10% goat serum, 0.1% Triton X-100 in PBS) for 2 h at 4 °C. Then, the slices were incubated with goat anti-Iba1 (1:1000, Abcam) in blocking solution overnight at 4 °C, followed by incubation with mouse anti-rabbit IgG Alexa Fluor 594 (1:200, Invitrogen) and mouse anti-goat IgG (1:200, Invitrogen) for 2 h at room temperature. All antibodies were diluted in PBS with 0.1% Triton X-100 and 2% bovine serum albumin. Nuclei were counterstained using Hoechst 33258. Slices were then coverslipped with 50% glycerin, and stained samples were photographed and analyzed using a FluoView FV1000 microscope (Olympus, Tokyo, Japan).

### Determination of glutamate and GABA levels

Mice were anesthetized with sodium pentobarbital and mounted on a stereotaxic frame (David Kopf Instruments, Tujunga, CA, USA). A microdialysis probe (CMA7 model, Carnegie Medicine, Stockholm, Sweden) was implanted in the left BLA nucleus (coordinates: − 1.45 mm anterior to the bregma, ± 2.5 mm lateral from the midline, and 4.3 mm beneath the surface of the skull). On the day of the experiment, the probe was perfused with artificial cerebrospinal fluid at a flow rate of 1.5 μL/min using a CMA/100 pump (Carnegie Medicine, Stockholm, Sweden). After the probe trial of the behavioral tests, following a 30-min equilibration period, dialysate samples were collected every 15 min for 60 min and stored immediately at − 80 °C. Only mice with correctly implanted probes were included in the data analysis.

Levels of glutamate and GABA in the BLA were detected by reverse-phase high-performance liquid chromatography (HPLC, Agilent Technologies 1260 Infinity, Santa Clara, CA, USA) according to previously reported methods [24, 25]. 2, 4- dinitrofluorobenzene (DNFB) was used for pre-column derivatization. Microdialysate samples (50 μL) were mixed with 50 μL 0.5 mol/L NaHCO_3_ solution and 100 μL DNFB for 1 h at 60 °C. Then, 300 μL phosphate buffer (pH 7.0) was added to stop the reaction. The resulting products were analyzed using a UV detector at an absorbance of 360 nm. The mobile phase was KH_2_PO_3_ buffer (0.05 mol/L, pH 6.0)-acetonitrile-H_2_O (84:8:8, v/v/v) at a flow rate of 1.0 mL/min. A Thermo TC-C18 column (4.6 × 250 mm^2^; particle size: 5 mm) was used. The concentrations were calculated using LCsolution software (Shimadzu, Kyoto, Japan) based on standard samples.

### Molecular docking analysis

Docking analyses of SP with GABA-T (PDB code: 1OHW), GABA_A_ receptor (GABA_A_R) (PDB code: 6HUP), N-methyl-D-aspartate (NMDA) receptor (NMDAR) (PDB code: 4PE5), and α-amino-3-hydroxy-5-methyl-4-isoxazolepropionic acid (AMPA) receptor (AMPAR) (PDB code: 6QKC) were performed using the Glide module of Maestro 11.9 [26, 27]. All protein structures were downloaded from the Protein Data Bank (http://www.rcsb.org) and prepared using the Protein Preparation Wizard Workflow in the Schrodinger suite [26]. This involved the addition of hydrogen atoms to the protein, the assignment of bond orders, and the deletion of unnecessary water molecules. Moreover, H-bonds were optimized, and in the end, restrained minimization was performed wherein the heavy atoms were converged to root mean square deviation 0.3 Å. SP and the original crystal ligands were sketched in a 3D format and prepared for docking using the Ligand Preparation Application in the Schrodinger suite. The Receptor Grid Generation Workflow was used to define a grid around the bound co-crystallized ligand, and the grid was then used for docking SP in the ligand-binding site. Extra Precision (XP) mode was used for the docking analyses. For the validation of the docking parameters, the co-crystal ligand was re-docked at the catalytic site of the protein. The ligand interaction tool was used to view the interaction diagram of the ligands with the residues at the active site of the target protein.

### Statistical analysis

Results are expressed as the mean ± standard error of the mean (SEM). Statistical analysis of multiple groups was performed using one-way analysis of variance (ANOVA) in Microsoft Excel and Prism (GraphPad, San Diego, CA, USA). In all cases, *p* < 0.05 was considered to be statistically significant.

## Results

### SP alleviates anxiety-like behaviors induced by CFA injection

**Fig. 1:**
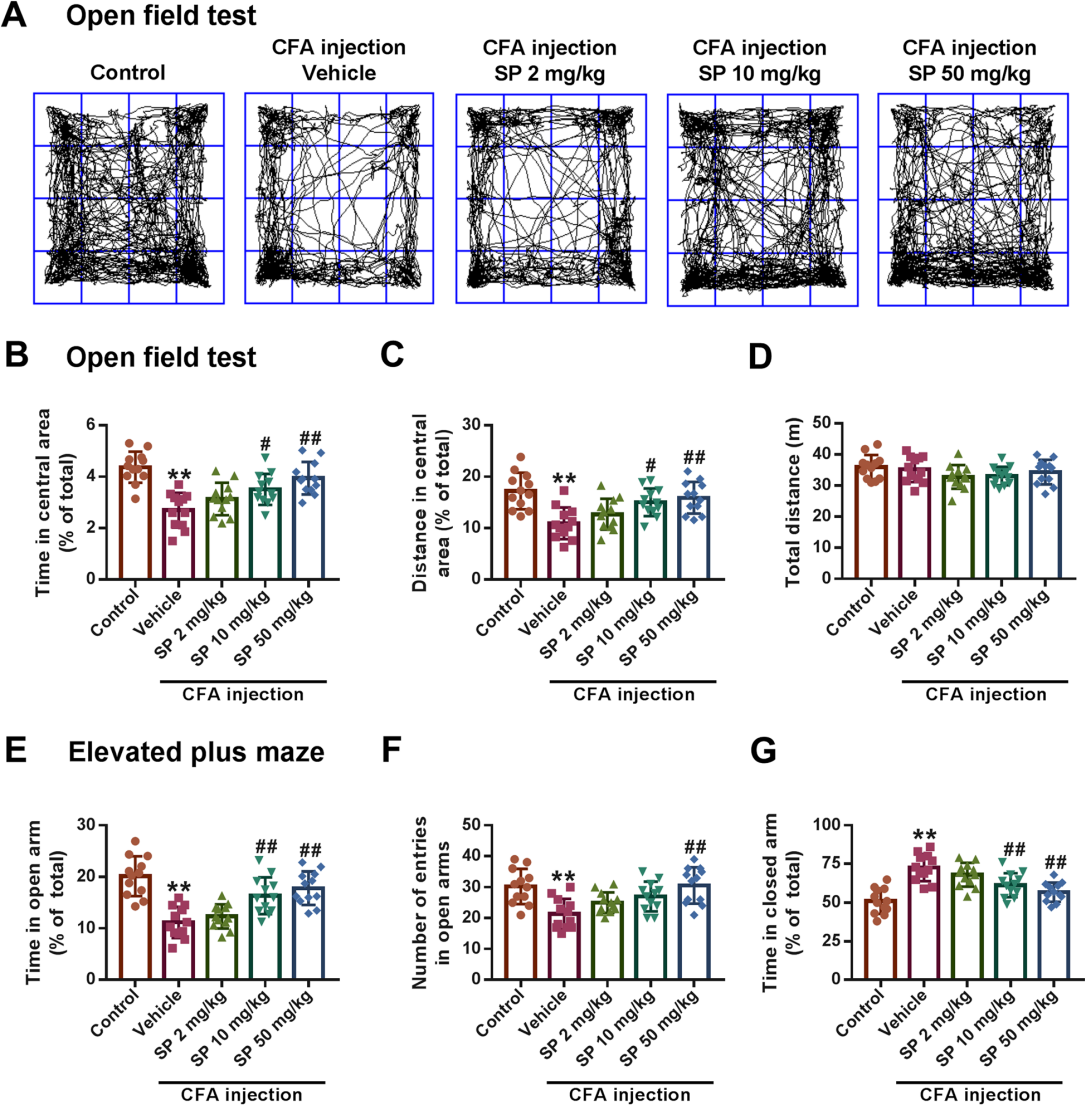
SP relieved CFA-induced anxiety-like behaviors in mice. **a** Representative traces of locomotor activity in the OFT. **b**-**d** SP administration effectively reversed the reduction in the time spent (**b**) and the distance traveled (**c**) in the central area in the OFT after CFA injection, while the total distance traveled showed no significant difference in each group (**d**). **e**-**g** SP treatment obviously increased the percentage of time spent in (**e**) and the number of entries into (**f**) the open arms, and decreased the percentage of time spent in (**g**) the closed arms in the EPM test. *n* = 12 mice per group; **p < 0.01 versus control group; ^#^*p* < 0.05, ^##^*p* < 0.01 versus vehicle group

The effects of SP on anxiety-like behaviors in mice were assessed using OFT and EPM tests. Compared with the control group, CFA-injected mice showed shorter times spent and distances traveled in the central area of the OFT (Fig. 1a-c), which indicates anxiety-like symptoms. Administration of SP dose-dependently blocked these changes (Fig. 1b and c). However, the total distance traveled showed no remarkable change in any group (Fig. 1d), suggesting that the mice had no deficit in locomotor activities. In the EPM test, decreased times spent in and lower numbers of entries into the open arms, as well as increased times spent in the closed arms, were observed after CFA injection. Similarly, treatment with SP could effectively reverse these effects (Fig. 1e-g). These results indicate anxiolytic effects of SP in CFA-injected mice.

### SP suppresses inflammation in the serum and the BLA of CFA-injected mice

**Fig. 2:**
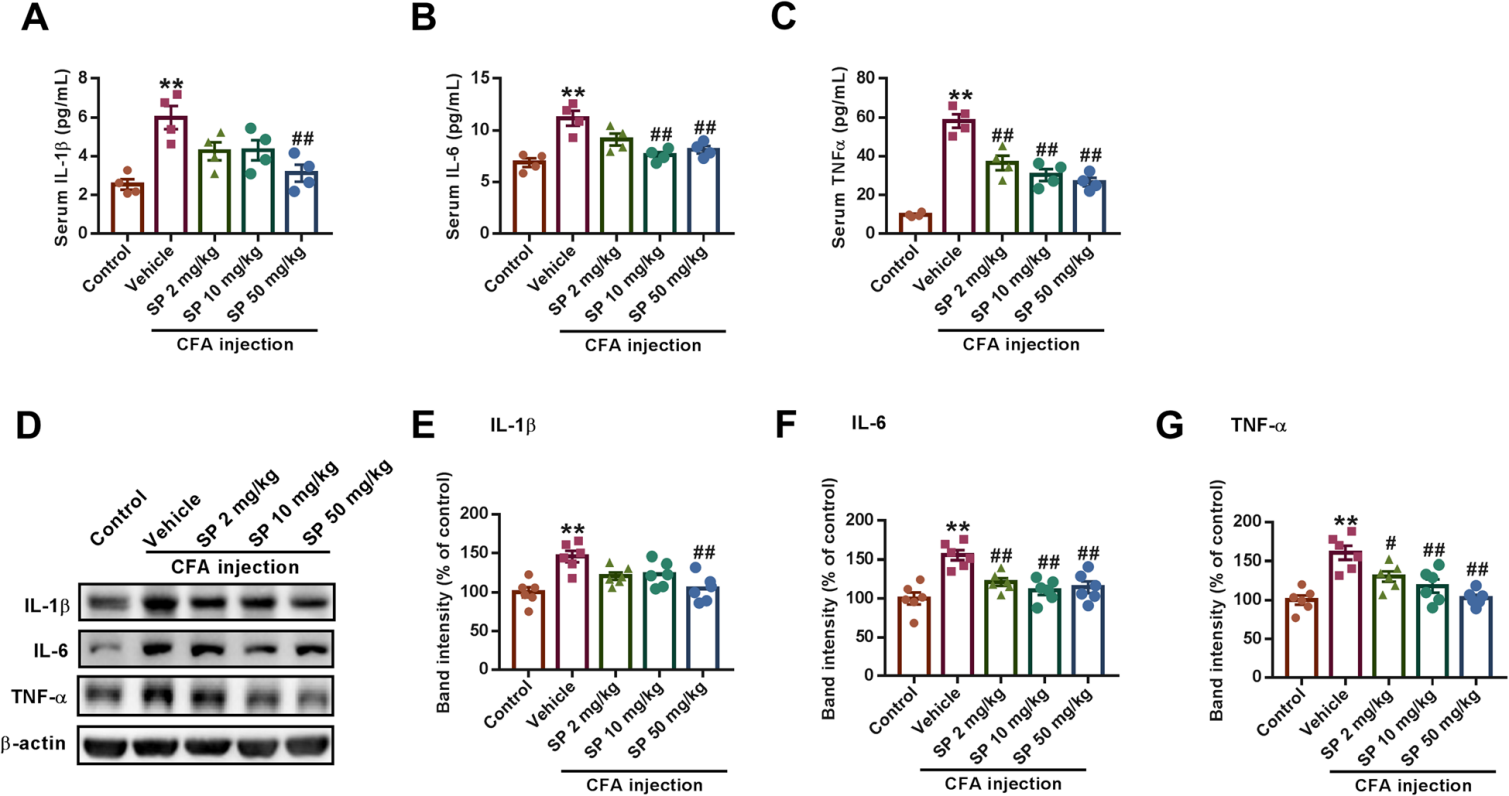
SP suppressed pro-inflammatory cytokine levels in the serum and BLA of CFA-injected mice. **a**-**c** SP treatment significantly reduced the elevated levels of IL-1β (**a**), IL-6 (**b**), and TNF-α (**c**) in the serum, as shown by ELISA. **d** Representative western blot analysis of IL-1β, IL-6, and TNF-α expression. Administration of SP reversed the increased expression of IL-1β (**e**), IL-6 (**f**), and TNF-α (**g**) normalized to β-actin. *n* = 6 mice from three independent experiments; **p < 0.01 versus control group; ^#^*p* < 0.05, ^##^*p* < 0.01 versus vehicle group

**Fig. 3:**
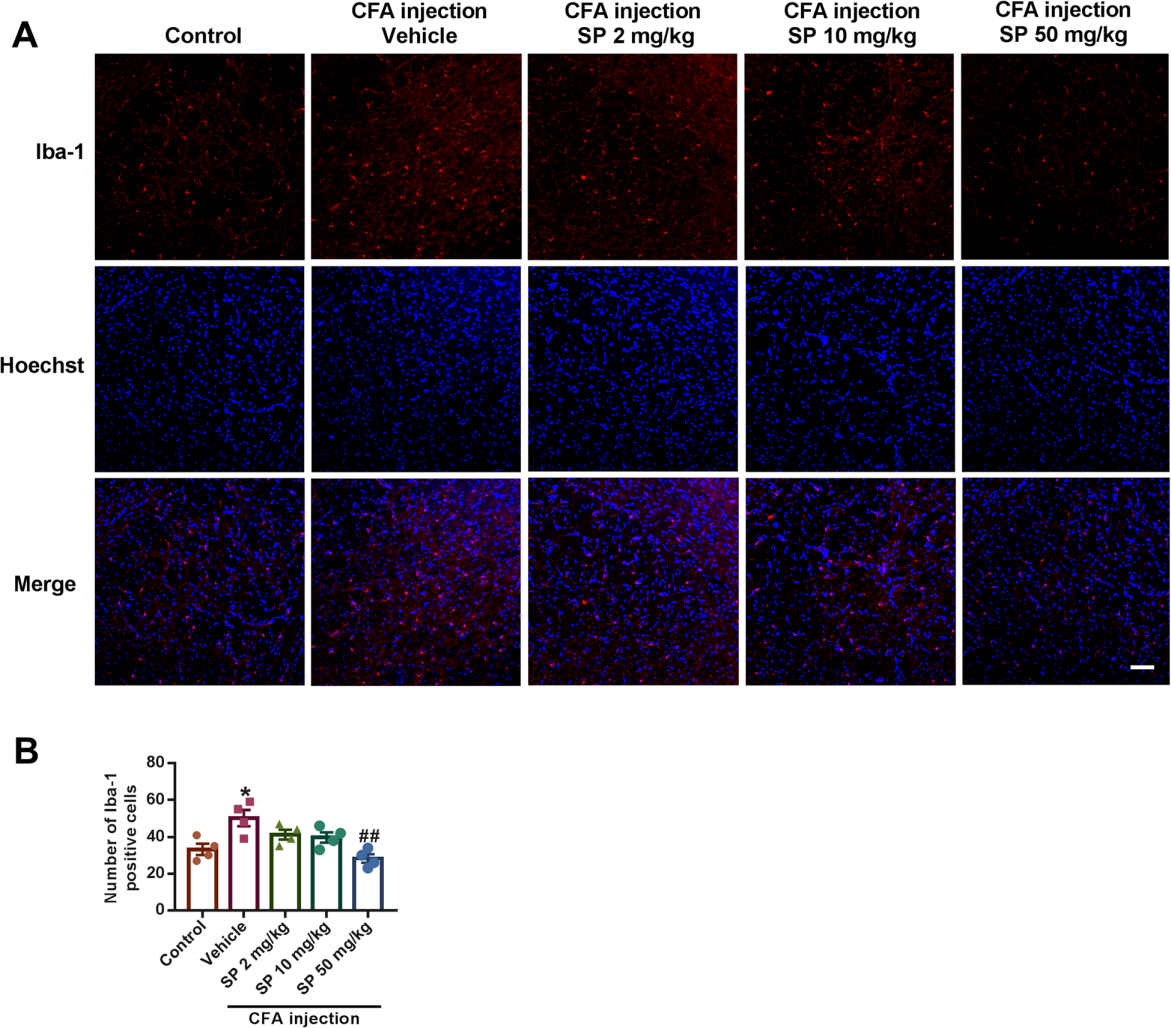
SP reduced microglial activation in the BLA of CFA-injected mice. **a** Slices were immunostained using the microglial marker Iba-1 antibody (red), and nuclei were stained with Hoechst 33258 (blue). Scale bar = 50 μm. **b** SP inhibited the activation of microglia in the BLA after CFA injection and had a dose-dependent effect. *n* = 6 mice from three independent experiments; *p < 0.05 versus control group; ^##^*p* < 0.01 versus vehicle group

Due to the important role of inflammation in the pathophysiology of anxiety, we measured the effects of SP on the levels of cytokines in the serum and the BLA. ELISA showed that CFA injection significantly elevated IL-1β, IL-6, and TNF-α levels in the serum (Fig. 2a-c). Similarly, the expression levels of these pro-inflammatory cytokines were also significantly elevated in the BLA of CFA-injected mice (Fig. 2d-g). SP treatment dose-dependently decreased the IL-1β, IL-6, and TNF-α levels (Fig. 2). As the inflammatory response is mainly mediated by microglia in the brain, we next tested the effect of SP on the activation of microglia. Immunofluorescence staining revealed that the number of Iba-1 positive cells was significantly increased in the BLA region of CFA-injected mice, suggesting that microglia were activated after CFA injection. SP administration decreased the number of activated microglia in the BLA (Fig. 3). Together, these data indicate that SP alleviates both peripheral and central inflammation.

### SP corrects the imbalance in excitatory/inhibitory receptors and neurotransmitters

**Fig. 4:**
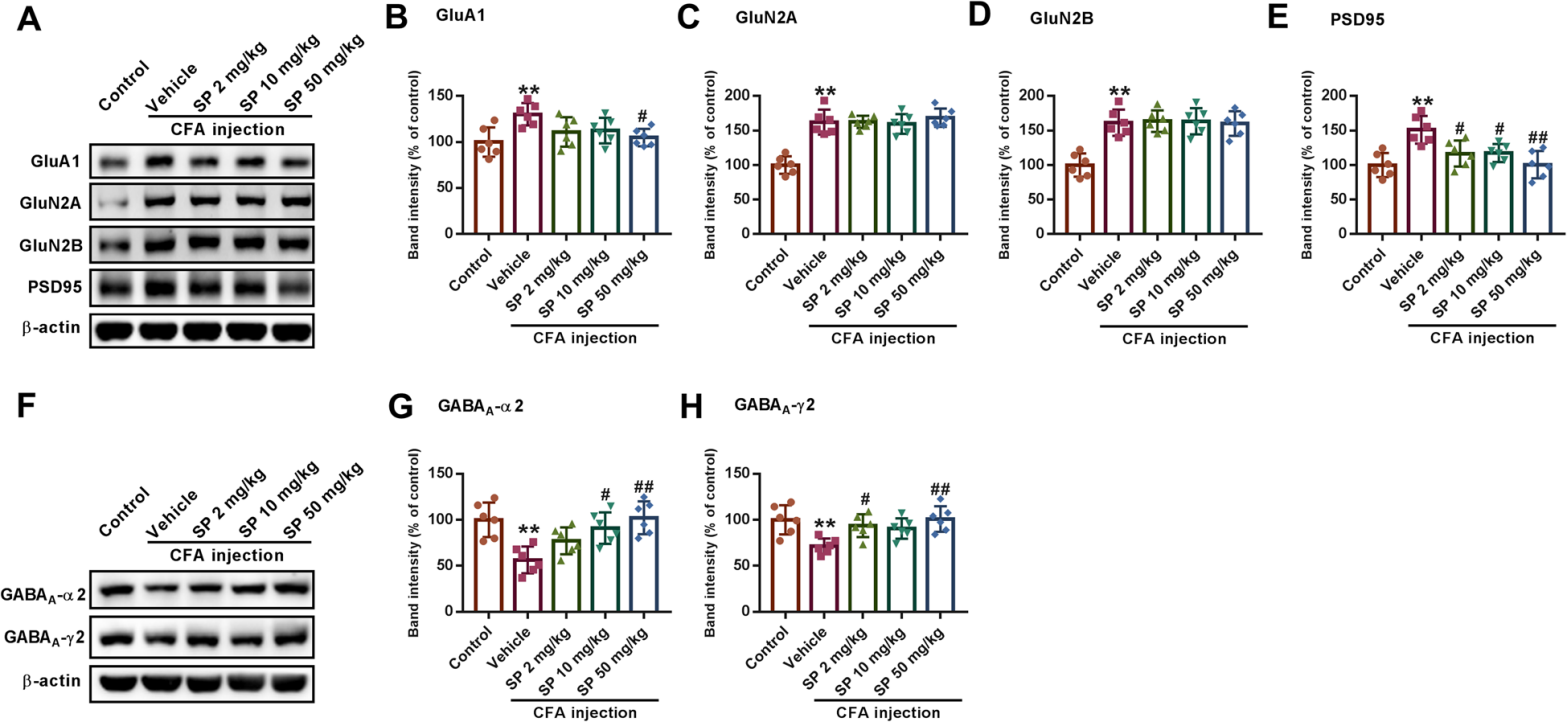
SP improved changes in glutamate and GABA_A_ receptor expression in the BLA of CFA-injected mice. **a** Representative western blot analysis of GluA1, GluN2A, GluN2B, and PSD95 expression. SP treatment reversed the increased expression of GluA1 (**b**) and PSD95 (**e**), but had no apparent effect on GluN2A (**c**) and GluN2B (**d**) normalized to β-actin. **f** Representative western blot analysis of GABA_A_ α2 and GABA_A_ γ2 expression. SP treatment significantly reversed the decreased expression of GABA_A_ α2 (**g**) and GABA_A_ γ2 (**h**) normalized to β-actin. *n* = 6 mice from three independent experiments; **p < 0.01 versus control group; ^#^*p* < 0.05, ^##^*p* < 0.01 versus vehicle group

The balance between excitatory and inhibitory transmission is critical for physiological anxiety, and prolonged disturbance of this balance can promote anxiety-like behaviors [28]. Hence, we first determined the expression alterations in excitatory AMPA and NMDA receptors, which play crucial roles in regulating synaptic neurotransmission and plasticity. We found that the expression levels of GluA1, GluN2A, GluN2B, and post-synaptic density protein-95 (PSD-95), another key protein involved in excitatory synaptic signaling, remarkably increased after CFA injection (Fig. 4a-e). Treatment with SP regulated the alteration of GluA1 and PSD95 in the BLA. However, there was no significant effect on the levels of GluN2A and GluN2B in CFA-injected mice after SP administration. Furthermore, GABA_A_ receptor-mediated inhibitory transmission in the BLA is also crucial for the development of anxiety. Therefore, the expression levels of GABA_A_ α2 and GABA_A_ γ2 subunits were examined. CFA induced a notable decrease in the expression of GABA_A_ α2 and GABA_A_ γ2, and this effect could be dose-dependently blocked by SP treatment (Fig. 4f-h). Therefore, the above results collectively suggest that SP can regulate the changes in excitatory and inhibitory synaptic receptors after CFA injection.

**Fig. 5:**
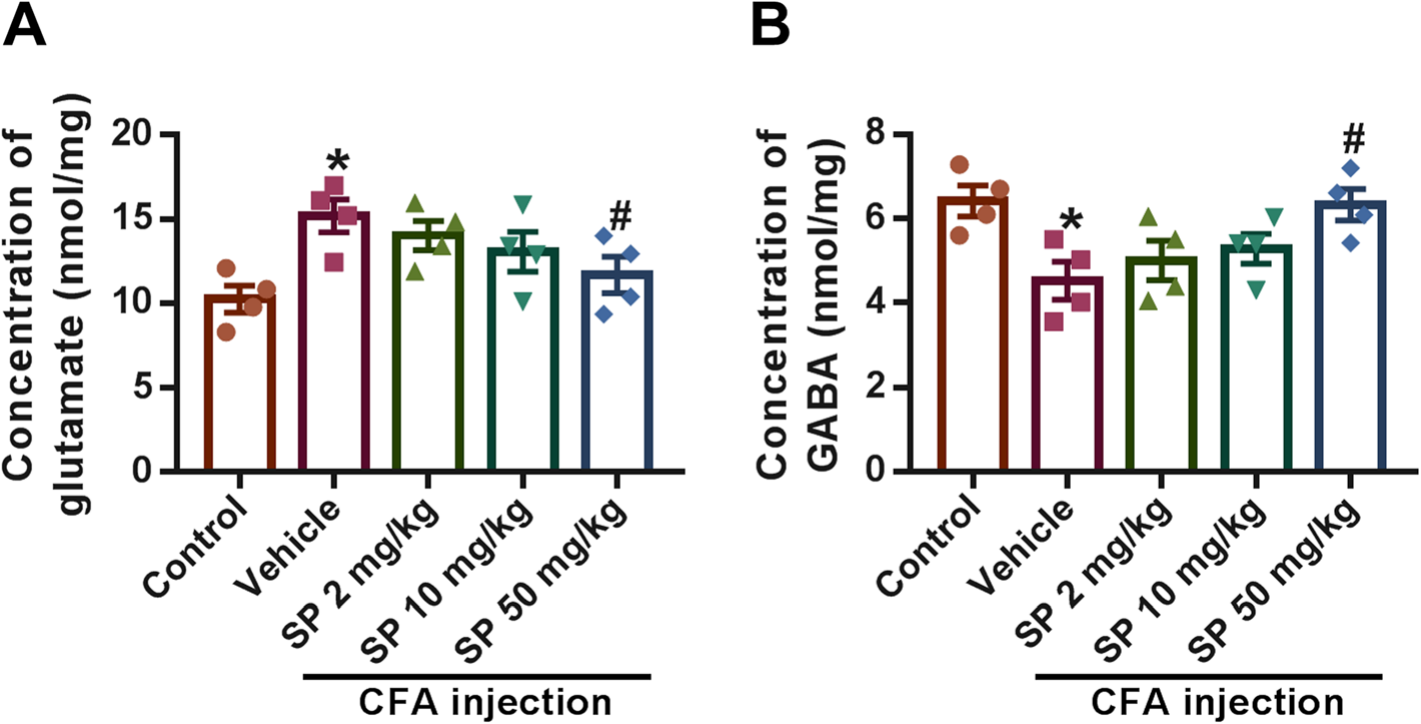
Effect of SP on glutamate and GABA levels in the BLA of CFA-injected mice. SP treatment reversed the increase in glutamate (**a**) and reduced GABA (**b**). Data are from three independent experiments; *p < 0.05 versus control group; ^#^*p* < 0.05 versus vehicle group

Glutamate and GABA are major excitatory and inhibitory neurotransmitters in the CNS. Hence, we subsequently measured the concentrations of glutamate and GABA in the BLA. Compared to the control group (glutamate: 10.240 ± 0.805 nmol/mg; GABA: 6.420 ± 0.365 nmol/mg), the CFA mice displayed higher glutamate (15.190 ± 0.984 nmol/mg) and lower GABA (4.522 ± 0.452 nmol/mg) levels, while this alteration could be significantly modulated by a high dose of SP (Fig. 5a and b). These results indicate that, besides postsynaptic receptors, the levels of glutamate and GABA are also regulated by SP in CFA-injected mice.

### SP regulates inflammation by inhibiting GABA-T and associated signals

**Fig. 6:**
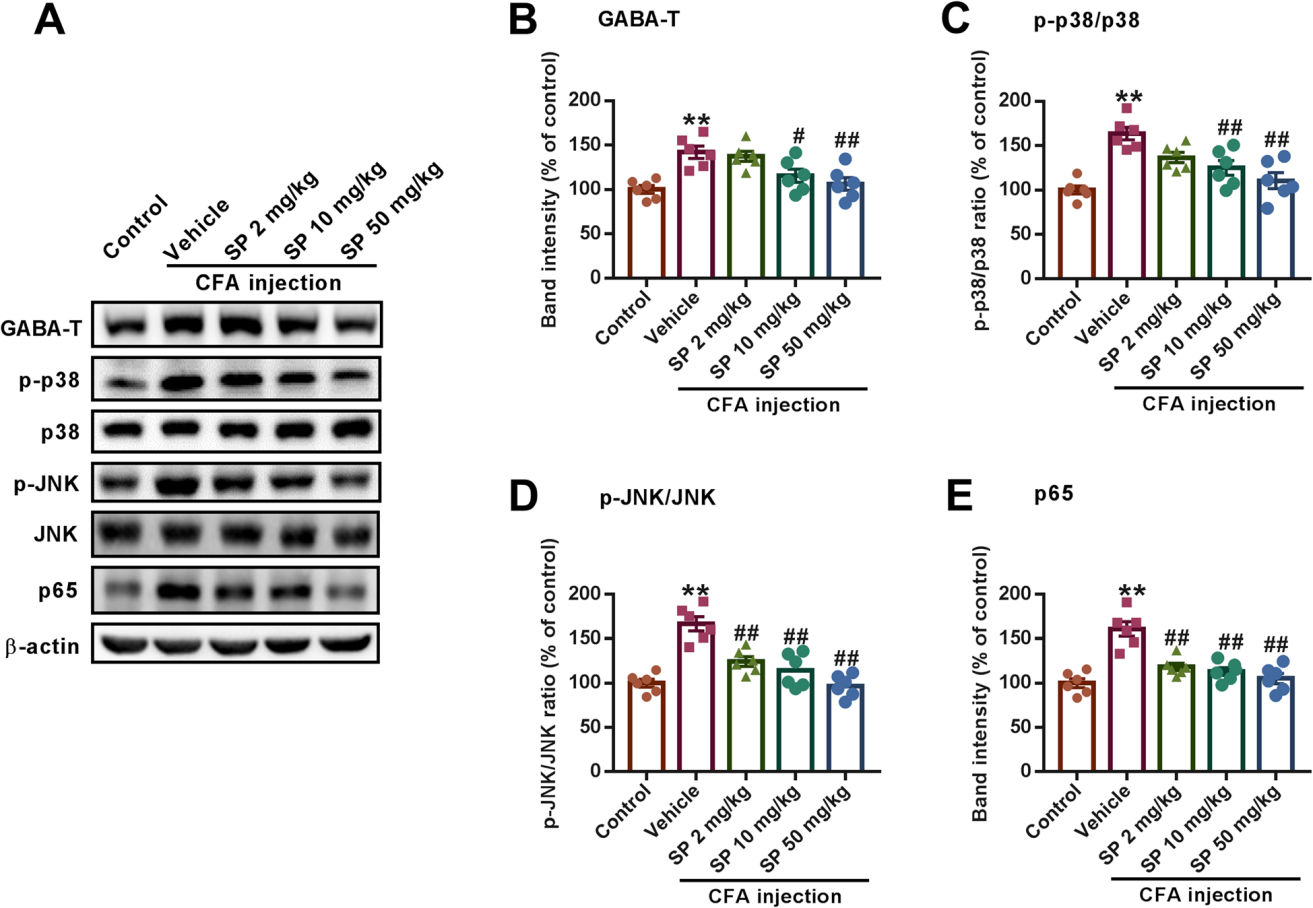
SP inhibited GABA-T and NF-κB and MAPK signaling pathways in CFA-induced mice. **a** Representative western blot analysis of GABA-T, p-p38, p38, p-JNK, JNK, and p65 expression. SP treatment obviously reduced the CFA-induced upregulations of GABA-T (**b**), p-p38/p38 (**c**), p-JNK/JNK (**d**), and p65 (**e**) normalized to β-actin. *n* = 6 mice from three independent experiments; **p < 0.01 versus control group; ^#^*p* < 0.05, ^##^*p* < 0.01 versus vehicle group

In order to find the reason for the dysregulation in neuronal transmission, we focused on the critical enzyme involved in GABA metabolism, GABA-T, which decreases the level of GABA in the brain while increasing the level of glutamate [29]. Therefore, we determined the expression levels of GABA-T in the BLA after CFA injection. We found that CFA resulted in increased levels of GABA-T, and SP administration blocked this change (Fig. 6a and b). Furthermore, studies have shown that GABA-T can regulate levels of inflammatory cytokines by associated signaling molecules [29]. The effect of SP on the NF-κB and MAPK signaling molecules was detected in the BLA after CFA injection. Western blot results showed that the phosphorylation levels of p38 and JNK and the expression levels of the NF-κB subunit p65 were obviously increased in CFA-induced mice, while treatment with different doses of SP effectively reversed these alterations (Fig. 6a and c-e). Collectively, these data suggest that SP can regulate inflammatory responses by inhibiting the NF-κB and MAPK signaling pathways in which GABA-T is involved.

### SP has high affinity to GABA-T and GABA_A_ receptor via molecular docking

**Fig. 7:**
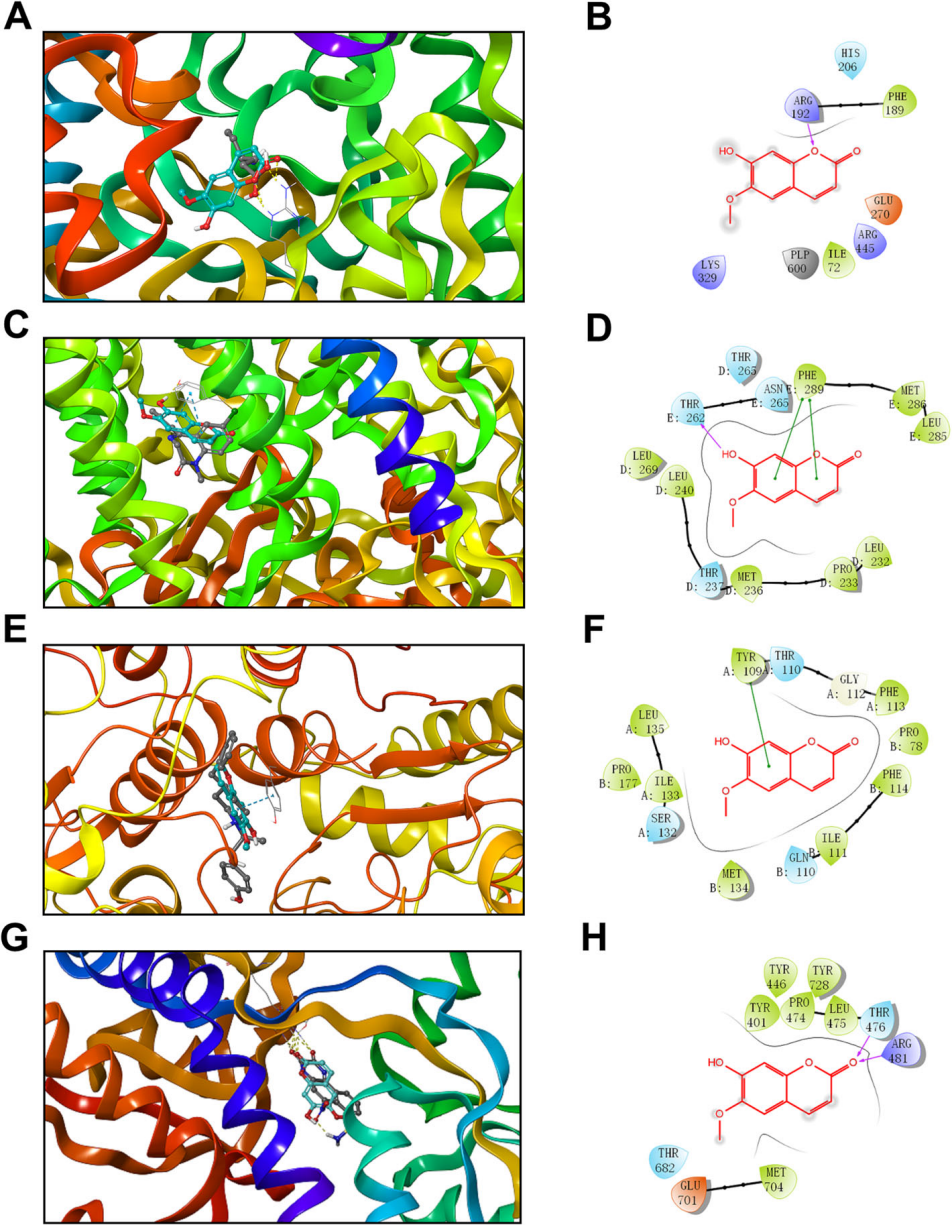
Binding interactions of SP with GABA-T, GABA_A_R, NMDAR, and AMPAR. **a-h** Superimposition of SP (gray) with the co-crystallized ligands (light blue) vigabatrin (**a**), diazepam (**c**), ifenprodil (**e**), and NBQX (**g**) against GABA-T (PDB code: 1OHW), GABA_A_R (PDB code: 6HUP), NMDAR (PDB code: 4PE5), and AMPAR (PDB code: 6QKC); the yellow and light blue dashes represent hydrogen bonds and π-π stacking, respectively. The 2D interaction diagram shows the major binding sites between SP and GABA-T (**b**), GABA_A_R (**d**), NMDAR (**f**), and AMPAR (**h**); the purple arrow and the green line represent hydrogen bonds and π-π stacking, respectively

To further investigate whether SP directly interacts with GABA-T, a molecular docking analysis of this compound was conducted. We found that SP bound tightly in the active site of GABA-T and formed a hydrogen bond with Arg192 and hydrophobic interactions with Phe189 and Tyr69 (Fig. 7a and b). The binding mode of SP was similar to that of the crystal ligand vigabatrin, which is clinically used in the treatment of epilepsy and also has a positive effect on anxiety [30, 31]. This suggests that GABA-T inhibition was responsible for the anti-anxiety effect of SP. In addition, SP also showed good affinity to the benzodiazepine (BZD) binding site of GABA_A_R. Docking results revealed that SP occupied the binding pocket of diazepam and made hydrophobic interactions with Leu285, Met286, Phe289, Leu240, Met236, Pro233, and Leu232. Moreover, the chromene moiety of SP could form π-π stacked interactions with Phe289, and a hydrogen bond between the OH group and Thr262 was also observed (Fig. 7c and d), suggesting that SP had higher affinity than diazepam [32]. We also investigated the interactions of SP with excitatory NMDA and AMPA receptors. However, the primary interactions between SP and NMDAR were π-π stacking with Tyr109 and hydrophobic interactions with several residues such as Phe113, Pro78, Phe114, and Met134 (Fig. 7e and f). As for AMPAR, the carbonyl oxygen of SP formed two hydrogen bonds with Arg481 and Thr476, respectively. The residues Met704, Leu475, Tyr728, Tyr446, Pro474, and Tyr401 showed hydrophobic interactions with SP (Fig. 7g and h). However, this effect appeared to be weaker than that of 2,3-dihydroxy-6-nitro-7-sulfamoyl-benzo [f] quinoxaline (NBQX), a competitive antagonist of AMPAR [33]. Therefore, SP is more likely to modulate the function of inhibitory synaptic receptors than of excitatory synaptic receptors.

### SP exerts anti-anxiety effects by activating GABA_A_ receptors

**Fig. 8:**
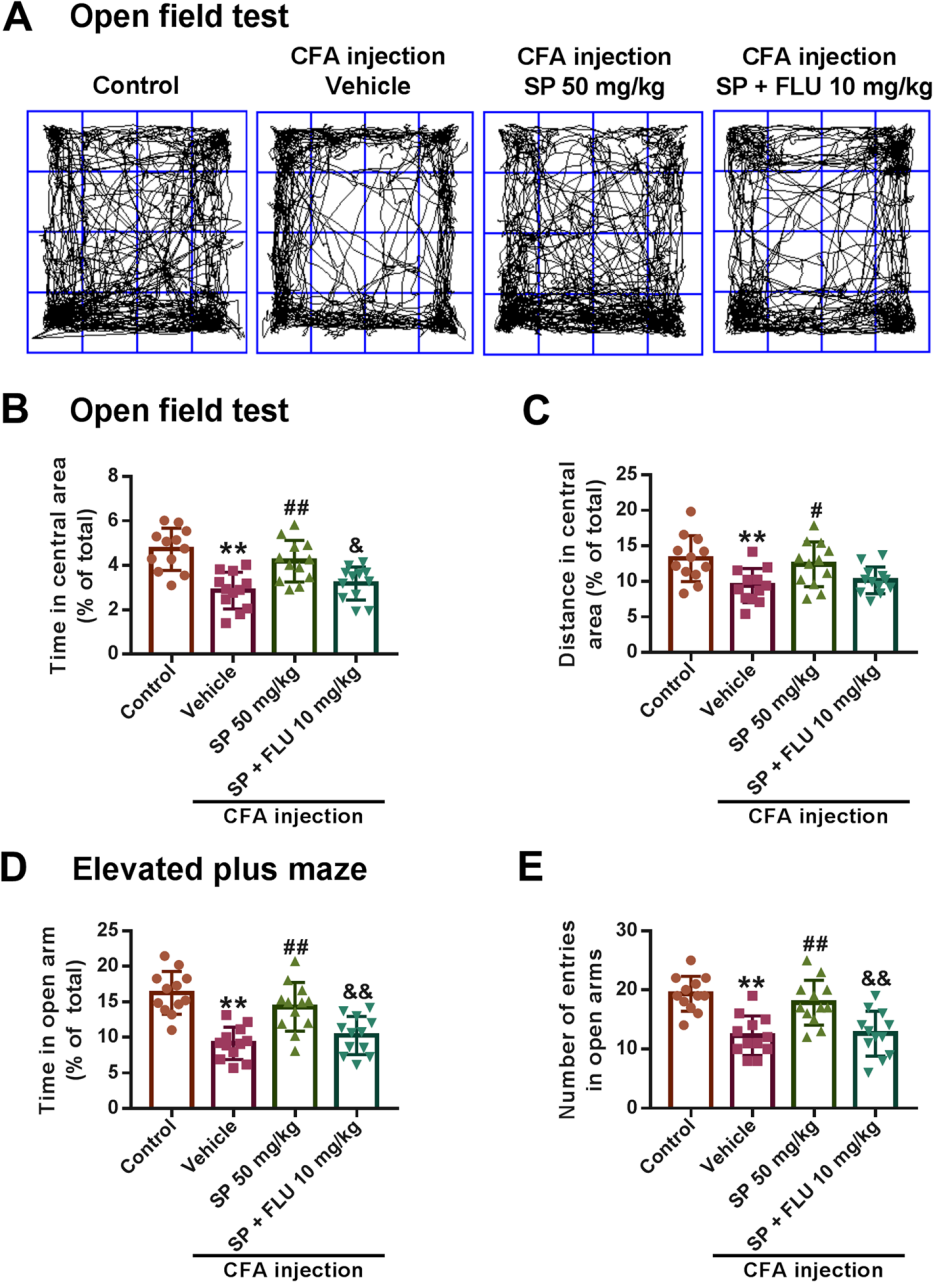
SP alleviated CFA-induced anxiety behaviors by activating the GABA_A_ receptor. **a** Representative traces of locomotor activity in the OFT. **b, c** SP (50 mg/kg) administration effectively reversed the reduction in the time spent (**b**) and distance traveled (**c**) in the central area in the OFT, while this effect was reduced by co-administration of flumazenil (FLU, 10 mg/kg). **d, e** Mice showed a significant increase in the percentage of time spent in (**d**) and number of entries into (**e**) the open arms in the EPM test after SP (50 mg/kg) treatment, while this effect of SP was also reduced in the presence of FLU (10 mg/kg). *n* = 12 mice per group; **p < 0.01 versus control group; ^#^*p* < 0.05, ^##^*p* < 0.01 versus vehicle group; ^&^*p* < 0.05, ^&&^*p* < 0.01 versus SP group

In order to further confirm that the inhibitory GABA_A_ receptor is involved in the anxiolytic actions of SP, the specific GABA_A_ receptor antagonist flumazenil (10 mg/kg) was used. Treatment with SP (50 mg/kg) consistently and significantly reversed the decrease in times spent and distances traveled in the central area induced by CFA injection in the OFT (Fig. 8a-c). However, in the presence of flumazenil (10 mg/kg), this effect of SP was reduced (Fig. 8b and c). In the EPM test, increased times spent in and higher numbers of entries into the open arms was observed in SP-treated mice. Similarly, the beneficial effect was attenuated by co-administration of flumazenil (Fig. 1d and e). Therefore, these results suggest that the GABA_A_ receptor plays an important role in the anxiolytic effects of SP.

## Discussion

As existing drug treatments are not effective for all patients, the search for new and better anxiolytic drugs to prevent and treat anxiety continues [34]. Here, we first showed that SP treatment effectively relieved CFA-induced anxiety-like behaviors in mice. In addition, we further discovered that the inhibition of inflammation and the regulation of the imbalance between excitatory and inhibitory transmission might be responsible for the anxiolytic actions of SP.

Several studies have demonstrated increased anxiety-like behavior in response to inflammation [5, 35]. Interest in the role of inflammation in mood and anxiety disorders has prompted research on the blockade of inflammation as a potential treatment strategy [36]. In our present study, both peripheral and central IL-1β, IL-6, and TNF-α levels were significantly increased after CFA injection, and the injected mice showed obvious anxiety-like behaviors. Treatment with SP could relieve anxiety-like symptoms in CFA-injected mice and reduce the levels of pro-inflammatory cytokines, which is consistent with previous findings showing that SP exerts anti-inflammatory effects. Moreover, previous studies have demonstrated that CFA injection activates the microglia, the primary inflammatory mediators and main source of cytokines in the CNS [23]. The same was observed in the current study, and SP was found to reduce CFA-induced microglial activation in the BLA. This suggests that SP can improve anxiety-like behaviors through its anti-inflammatory effects.

GABA is the major inhibitory neurotransmitter in the mammalian CNS and plays a key role in normal brain function. GABA has been shown to suppress the reactive response of both astrocytes and microglia to inflammatory LPS stimulants and to result in a reduced release of the inflammatory cytokines TNF-α and IL-6 [29]. Therefore, GABA may be involved in the anti-inflammatory effect of SP. GABA is metabolized by GABA-T, a mitochondrial enzyme that decreases the level of GABA in the brain while increasing the level of glutamate. Enhanced glutamate excitatory transmission and reduced inhibitory GABA transmission have been associated with inflammation, and have been shown to cause hyperexcitation that promotes pathological anxiety-like behaviors [37, 38]. These earlier studies suggested that the regulation of glutamate and GABA levels is important in anxiety. A previous study has also revealed that SP can significantly inhibit GABA-T [19]. We found good affinity of SP to GABA-T via molecular docking and thus hypothesized that SP might regulate the levels of glutamate and GABA by suppressing GABA-T, thereby relieving anxiety in CFA mice. To confirm this, we further investigated GABA-T expression and glutamate and GABA concentrations in CFA-injected mice after SP treatment. As expected, western blot analysis showed that SP reduced GABA-T expression, and HPLC demonstrated that SP changed the levels of glutamate and GABA in the BLA, with the former decreased and the latter increased. Indeed, GABA-T inhibitors such as vigabatrin are clinically used in the treatment of epilepsy and have a positive effect on anxiety. Overall, restoring the imbalance between excitatory and inhibitory neurotransmitters might be responsible for the anxiolytic actions of SP, and GABA-T could act as a target for SP.

Given that glutamate and GABA act on their corresponding receptors, we next determined the expression of excitatory glutamate receptors and inhibitory GABA receptors during CFA-induced anxiety. Ionotropic glutamate receptors include AMPAR and NMDAR, such as GluA1, GluA2, GluN2A, and GluN2B, which play critical roles in regulating synaptic neurotransmission and plasticity as well as in anxiety [39]. In this study, CFA resulted in increased expression of GluA1, GluN2A, GluN2B, and PSD95, a postsynaptic anchor protein that binds to AMPA and NMDA receptors [40]. Treatment with SP down-regulated GluA1 and PSD95, but had no obvious effect on the levels of GluN2A and GluN2B. Our molecular docking analyses show that SP interacts weakly with the NMDA receptor, suggesting that SP treatment fails to regulate the excitatory NMDA receptor changes after CFA injection. Additionally, excitatory activity in the BLA is tightly regulated by a relatively small population of GABA-inhibitory neurons [41]. Accumulating evidence suggests that the GABA_A_ receptor subunits GABA_A_ α2 and GABA_A_ γ2 mediate anxiety in the BLA [42, 43]. Consistently, evident reductions in the levels of GABA_A_ α2 and GABA_A_ γ2 were observed in CFA mice in the current study. SP significantly reversed the expression of the inhibitory GABA_A_ receptors in the BLA. Moreover, there was a good affinity between SP and GABA_A_ receptors, which further suggested that the up-regulation of GABA_A_ receptors by SP was related to its binding ability. These results collectively indicate that the imbalance between enhanced excitatory and attenuated inhibitory synaptic transmission may be related to the changes in these receptors and neurotransmitters after CFA injection, which can be modified by SP, and suggest that the inhibitory GABA_A_ receptor might play a leading role in this process. This was further confirmed subsequently in the current work, where we found that co-administration of SP and a GABA_A_ receptor antagonist attenuated the positive effect of SP alone on the anxiety-like behaviors induced by CFA injection. These results thus indicate that the inhibitory GABA_A_ receptor, which is a crucial drug target for anxiolytics such as benzodiazepines, is also tightly involved in the anxiolytic actions of SP.

While our results show that the anti-inflammation and excitatory/inhibitory transmission balance is involved in the anti-anxiety effects of SP, the relationship between the two remains unclear. NF-κB regulates the expression of a wide variety of genes that play critical roles in inflammatory responses. These NF-κB target genes include those encoding cytokines (e.g., IL-1β, TNF-α, IL-6) [44]. MAPK molecules also play an important role by triggering a cascade reaction and ultimately resulting in expression of specific cellular genes encoding pro-inflammatory mediators [45]. Therefore, the expression of pro-inflammatory mediators is modulated by the NF-κB and MAPK pathways [46], which play a key role in the regulation of anxiety behavior [47, 48]. Treatment with NF-κB and MAPK inhibitors might thus have a beneficial effect on brain inflammation-induced anxiety and depression. Moreover, studies have shown that GABA can regulate the levels of inflammatory cytokines through the NF-κB and p38 MAPK pathways, and this effect is tightly linked with GABA-T, which modulates the imbalance between glutamate and GABA neurotransmitters. These data together provide a preliminary indication of a link between anxiety associated with an excitatory/inhibitory imbalance and inflammation mediated by the NF-κB and MAPK pathways, and GABA-T might play an important role in these processes. Therefore, based on the inhibitory effect of SP on GABA-T in this study, we further investigated the expression levels of NF-κB and MAPK molecules. Scopoletin has been reported to down-regulate gene transcription and production of the pro-inflammatory mediators, possibly by preventing the activation of the canonical NF-κB pathway and the phosphorylation of MAPK. Moreover, scopoletin suppresses p-JNK and p-p38 MAPKs, which may act alone or relate to the activation of NF-κB [18]. Our previous studies have shown that the expression of NF-κB p65, p-p38, and p-JNK MAPKs is increased in mice with chronic inflammation induced by CFA [23, 49]. These alterations were consistently confirmed in the present work, while SP treatment effectively reversed these alterations. These findings, in line with previous reports, thus indicate that the anxiolytic effects of SP are associated with the inhibition of the NF-κB and MAPK signaling pathways.

To conclude, the present results show that SP ameliorates anxiety-like behaviors induced by CFA injection in mice. Our findings suggest that the prevention of the NF-κB and MAPK signaling pathways involving anti-inflammatory activities and regulation of the excitatory/inhibitory balance attributes to the anti-anxiety effects of SP. Further studies need to assess whether SP exerts anxiolytic effects in other anxiety models, such as stress-induced and social anxiety models. In short, SP should be considered as a potential agent for further development in the treatment of anxiety, and other mechanisms involved in the processes described here should be investigated to offer some new targets for anti-anxiety drug research.

## Data Availability

The datasets supporting the conclusion of this study are included within the article.

## Abbreviations

BLA: Basolateral
BZD: Benzodiazepine
CFA: Complete Freund’s adjuvant
CNS: Central nervous system
DMSO: Dimethyl sulfoxide
DNFB: Dinitrofluorobenzene
EPM: Elevated plus maze
GABA-T: GABA transaminase
LPS: Lipopolysaccharides
MAPK: Mitogen-activated protein kinase
NBQX: 2,3-dihydroxy-6-nitro-7-sulfamoyl-benzo [f]quinoxaline
NF-κB: Nuclear factor-kappa B
OFT: Open field test
PSD-95: Post-synaptic density protein-95
RMSD: Root mean square deviation
SP: Scopoletin

## References

[1] Savage K, Firth J, Stough C, Sarris J (2018). GABA-modulating phytomedicines for anxiety: a systematic review of preclinical and clinical evidence. Phytother Res.

[2] Combs H, Markman J (2014). Anxiety disorders in primary care. Med Clin North Am.

[3] Offidani E, Guidi J, Tomba E, Fava GA (2013). Efficacy and tolerability of benzodiazepines versus antidepressants in anxiety disorders: a systematic review and meta-analysis. Psychother Psychosom.

[4] Rudolph U, Knoflach F (2011). Beyond classical benzodiazepines: novel therapeutic potential of GABAA receptor subtypes. Nat Rev Drug Discov.

[5] Felger JC (2018). Imaging the role of inflammation in mood and anxiety-related disorders. Curr Neuropharmacol.

[6] Lasselin J, Elsenbruch S, Lekander M, Axelsson J, Karshikoff B, Grigoleit JS (2016). Mood disturbance during experimental endotoxemia: predictors of state anxiety as a psychological component of sickness behavior. Brain Behav Immun.

[7] Muscatell KA, Dedovic K, Slavich GM, Jarcho MR, Breen EC, Bower JE (2015). Greater amygdala activity and dorsomedial prefrontal-amygdala coupling are associated with enhanced inflammatory responses to stress. Brain Behav Immun.

[8] Redlich R, Stacey D, Opel N, Grotegerd D, Dohm K, Kugel H (2015). Evidence of an IFN-γ by early life stress interaction in the regulation of amygdala reactivity to emotional stimuli. Psychoneuroendocrinology.

[9] Babaev O, Chatain CP, Krueger-Burg D (2018). Inhibition in the amygdala anxiety circuitry. Exp Mol Med.

[10] Prager EM, Bergstrom HC, Wynn GH, Braga MF (2016). The basolateral amygdala γ-aminobutyric acidergic system in health and disease. J Neurosci Res.

[11] Pearl PL, Gibson KM (2004). Clinical aspects of the disorders of GABA metabolism in children. Curr Opin Neurol.

[12] Wang KT, Liu HT, Chen XG, Yunkun Z, Zhide H (2001). Identification and determination of active components in *Angelica dahurica* Benth and its medicinal preparation by capillary electrophoresis. Talanta.

[13] Shaw CY, Chen CH, Hsu CC, Chen CC, Tsai YC (2003). Antioxidant properties of scopoletin isolated from *Sinomonium acutum*. Phytother Res.

[14] Lee SH, Ding Y, Yan XT, Kim YH, Jang HD (2013). Scopoletin and scopolin isolated from *Artemisia iwayomogi* suppress differentiation of osteoclastic macrophage RAW 264.7 cells by scavenging reactive oxygen species. J Nat Prod.

[15] Chen Z, Liao L, Zhang Z, Wu L, Wang Z (2013). Comparison of active constituents, acute toxicity, anti-nociceptive and anti-inflammatory activities of *Porana sinensis* Hemsl., *Erycibe obtusifolia* Benth. and *Erycibe schmidtii* Craib. J Ethnopharmacol.

[16] Chang Tien-Ning, Deng Jeng-Shyan, Chang Yi-Chih, Lee Chao-Ying, Jung-Chun Liao, Lee Min-Min, Peng Wen Huang, Huang Shyh-Shyun, Huang Guan-Jhong (2012). Ameliorative Effects of Scopoletin fromCrossostephium chinensisagainst Inflammation Pain and Its Mechanisms in Mice. Evidence-Based Complementary and Alternative Medicine.

[17] Kim HJ, Jang SI, Kim YJ, Chung HT, Yun YG, Kang TH (2004). Scopoletin suppresses pro-inflammatory cytokines and PGE_2_ from LPS-stimulated cell line, RAW 264.7 cells. Fitoterapia.

[18] Yao XJ, Ding ZQ, Xia YF, Wei Z, Luo Y, Feleder C (2012). Inhibition of monosodium urate crystal-induced inflammation by scopoletin and underlying mechanisms. Int Immunopharmacol.

[19] Mishra N, Oraon A, Dev A, Jayaprakash V, Basu A, Pattnaik AK (2010). Anticonvulsant activity of *Benkara malabarica* (Linn.) root extract: in vitro and in vivo investigation. J Ethnopharmacol.

[20] Capra JC, Cunha MP, Machado DG, Zomkowski AD, Mendes BG, Santos AR (2010). Antidepressant-like effect of scopoletin, a coumarin isolated from *Polygala sabulosa* (Polygalaceae) in mice: evidence for the involvement of monoaminergic systems. Eur J Pharmacol.

[21] Schmidt-Mutter C, Pain L, Sandner G, Gobaille S, Maitre M (1998). The anxiolytic effect of γ-hydroxybutyrate in the elevated plus maze is reversed by the benzodiazepine receptor antagonist, flumazenil. Eur J Pharmacol.

[22] Yue J, Wang XS, Guo YY, Zheng KY, Liu HY, Hu LN (2018). Anxiolytic effect of CPEB1 knockdown on the amygdala of a mouse model of inflammatory pain. Brain Res Bull.

[23] Fan YF, Guan SY, Luo L, Li YJ, Yang L, Zhou XX (2018). Tetrahydroxystilbene glucoside relieves the chronic inflammatory pain by inhibiting neuronal apoptosis, microglia activation, and GluN2B overexpression in anterior cingulate cortex. Mol Pain.

[24] Wang L, Wang J, Yang L, Zhou SM, Guan SY, Yang LK (2017). Effect of Praeruptorin C on 3-nitropropionic acid induced Huntington’s disease-like symptoms in mice. Biomed Pharmacother.

[25] Wang MM, Du WC, Shen J, Dong Y, Wei WS, Song ZJ (2013). Determination of amino acid neurotransmitters in mouse brain tissue using high-performance liquid chromatography with fluorescence detection. J Chin Pharm Sci.

[26] Iqubal A, Sharma S, Sharma K, Bhavsar A, Hussain I, Iqubal MK (2018). Intranasally administered pitavastatin ameliorates pentylenetetrazol-induced neuroinflammation, oxidative stress and cognitive dysfunction. Life Sci.

[27] Kumar S, Chowdhury S, Kumar S (2017). In silico repurposing of antipsychotic drugs for Alzheimer’s disease. BMC Neurosci.

[28] Wu LJ, Kim SS, Zhuo M (2008). Molecular targets of anxiety: from membrane to nucleus. Neurochem Res.

[29] Lee M, Schwab C, McGeer PL (2011). Astrocytes are GABAergic cells that modulate microglial activity. Glia.

[30] Storici P, De Biase D, Bossa F, Bruno S, Mozzarelli A, Peneff C (2004). Structures of γ-aminobutyric acid (GABA) aminotransferase, a pyridoxal 5′-phosphate, and [2Fe-2S] cluster-containing enzyme, complexed with γ-ethynyl-GABA and with the antiepilepsy drug vigabatrin. J Biol Chem.

[31] Sherif F, Harro J, EL-Hwuegi A, Oreland L (1994). Anxiolytic-like effect of the GABA-transaminase inhibitor vigabatrin (gamma-vinyl GABA) on rat exploratory activity. Pharmacol Biochem Behav.

[32] Masiulis S, Desai R, Uchański T (2019). GABA_A_ receptor signalling mechanisms revealed by structural pharmacology. Nature.

[33] Herguedas B, Watson JF, Ho H, Cais O, García-Nafría J, Greger IH (2019). Architecture of the heteromeric GluA1/2 AMPA receptor in complex with the auxiliary subunit TARP γ8. Science.

[34] Monsef-Esfahani HR, Amini M, Goodarzi N, Saiedmohammadi F, Hajiaghaee R, Faramarzi MA (2013). Coumarin compounds of *Biebersteinia multifida* roots show potential anxiolytic effects in mice. DARU.

[35] Prager G, Hadamitzky M, Engler A, Doenlen R, Wirth T, Pacheco-López G (2013). Amygdaloid signature of peripheral immune activation by bacterial lipopolysaccharide or staphylococcal enterotoxin B. J NeuroImmune Pharmacol.

[36] Miller AH, Haroon E, Felger JC (2017). Therapeutic implications of brain-immune interactions: treatment in translation. Neuropsychopharmacology.

[37] Carmans S, Hendriks JA, Slaets H, Thewissen K, Stinissen P, Rigo JM (2013). Systemic treatment with the inhibitory neurotransmitter gamma-aminobutyric acid aggravates experimental autoimmune encephalomyelitis by affecting proinflammatory immune responses. J Neuroimmunol.

[38] Jiang JX, Yu Y, Kinjo ER, Du Y, Nguyen HP, Dingledine R (2019). Suppressing pro-inflammatory prostaglandin signaling attenuates excitotoxicity-associated neuronal inflammation and injury. Neuropharmacology.

[39] Popoli M, Yan Z, McEwen BS, Sanacora G (2011). The stressed synapse: the impact of stress and glucocorticoids on glutamate transmission. Nat Rev Neurosci.

[40] Wang XS, Guan SY, Liu A, Yue J, Hu LN, Zhang K (2019). Anxiolytic effects of Formononetin in an inflammatory pain mouse model. Mol Brain.

[41] Lucas EK, Clem RL (2018). GABAergic interneurons: the orchestra or the conductor in fear learning and memory?. Brain Res Bull.

[42] Choi YM, Kim KH (2015). Etifoxine for pain patients with anxiety. Korean J Pain.

[43] Jucaite A, Cselenyi Z, Lappalainen J, McCarthy DJ, Lee CM, Nyberg S (2017). GABA_A_ receptor occupancy by subtype selective GABA_Aα2,3_ modulators: PET studies in humans. Psychopharmacology.

[44] Pahl HL (1999). Activators and target genes of Rel/NF-kappaB transcription factors. Oncogene.

[45] Edmunds JW, Mahadevan LC (2004). MAP kinases as structural adaptors and enzymatic activators in transcription complexes. J Cell Sci.

[46] Fu YH, Liu B, Zhang NS, Liu Z, Liang D, Li F (2013). Magnolol inhibits lipopolysaccharide-induced inflammatory response by interfering with TLR4 mediated NF-κB and MAPKs signaling pathways. J Ethnopharmacol.

[47] Bortolotto V, Cuccurazzu B, Canonico PL, Grilli M (2014). NF-κB mediated regulation of adult hippocampal neurogenesis: relevance to mood disorders and antidepressant activity. Biomed Res Int.

[48] Wefers B, Hitz C, Hölter SM, Trümbach D, Hansen J, Weber P (2012). MAPK signaling determines anxiety in the juvenile mouse brain but depression-like behavior in adults. PLoS One.

[49] Sun T, Wang J, Li X, Li YJ, Feng D, Shi WL (2016). Gastrodin relieved complete Freund's adjuvant-induced spontaneous pain by inhibiting inflammatory response. Int Immunopharmacol.

